# Experimental Study on Ocular Surface Protection by Soft Contact Lenses Due to Volcanic Ash Exposure

**DOI:** 10.3390/jcm13175281

**Published:** 2024-09-06

**Authors:** Hiroshi Toshida, Yusuke Matsuzaki, Masahiro Miyazaki

**Affiliations:** 1 Department of Ophthalmology, Juntendo University Shizuoka Hospital, 1129 Nagaoka, Izunokuni 410-2295, Japan; 2 Shizuoka Research Center for Disaster Medicine, Juntendo University, Izunokuni 410-2295, Japan

**Keywords:** contact lens, soft contact lens, volcanic ash, ashfall, eye, cornea, conjunctiva, injury, disaster

## Abstract

**Background**: Sudden volcanic eruptions can lead to volcanic ash entering the eyes, causing severe discomfort and complicating evacuation efforts. The specific effects of volcanic ash on ocular tissues, especially when wearing soft contact lenses (SCLs), are not well documented, prompting this experimental investigation. **Methods**: White rabbits with normal eyes were randomly divided into three groups: (1) a bare eye group: bare eye + volcanic ash exposure + eye washing, (2) an SCL group: SCL-wearing eye + volcanic ash exposure + eye washing, and (3) a control group: eye washing only. In groups 1 and 2, volcanic ash was applied to one eye under topical anesthesia, followed by immediate saline rinsing. Slit-lamp microscopy and histopathological analysis were conducted after euthanasia. **Results**: Slit-lamp and histopathological examinations revealed more significant corneal and conjunctival erosion in the bare eye group compared to the SCL group, which showed limited damage. The control group displayed no ocular damage. **Conclusions**: Guidelines from the “Volcanic Ash Health Effects: A Guide for the Public” by the National Research Institute for Earth Science and Disaster Resilience recommend removing SCLs during ashfall. Our findings suggest that the damage to the corneal and conjunctival epithelium is less severe in SCL-wearing eyes than in bare eyes, recommending that SCL wearers prioritize evacuation over lens removal during sudden ashfall.

## 1. Introduction

There are approximately 1300 active volcanoes on Earth [1]. To mitigate the health hazards posed by volcanic ash, the International Volcanic Health Hazard Network (IVHHN) actively engages in various initiatives [2]. One of these initiatives includes the compilation and publication of the “Volcanic Ash Health Effects: A Guide for the Public” [3]. This guide primarily addresses the direct adverse effects on human health, such as respiratory, ocular, and skin-related issues, caused by ashfall, as well as the environmental impacts and countermeasures related to roads, electricity, water supply, sanitation facilities, sewage treatment plants, roof collapses, and the effects on animals.

Regarding the use of contact lenses (CLs), the guide offers advice under the section “What to do to protect yourself against ash”, recommending the following: “In fine-ash environments, wear goggles or corrective eyeglasses instead of CLs to protect eyes from irritation” [3]. While this recommendation is effective for preparation and taking precautions before entering ash-affected areas, it may not be practical in situations requiring an immediate response, such as during a sudden eruption or phreatic explosion. For example, CL wearers are advised to remove their lenses during ashfall, but in scenarios where they must quickly evacuate due to an unexpected eruption or phreatic explosion, removing the lenses may not be feasible. This is because, without the lenses, their impaired vision could hinder their ability to escape safely.

Soft CLs (SCLs), currently used by approximately 94.5% of CL wearers [4], cover a large area of the ocular surface, including the entire cornea and part of the bulbar conjunctiva. Due to this extensive coverage, SCLs are sometimes used to protect or treat the eye, improving epithelial healing and reducing pain in persistent epithelial defects, after trauma or surgery, and in cases of corneal dystrophies [5]. Moreover, previous reports on the safety and efficacy of SCL wear in industrial and chemical workplaces have shown that CLs typically provide protective benefits that reduce the severity of ocular injury and improve worker performance, although contraindications for CL wear do exist [6,7]. Therefore, we conducted a study to evaluate the extent of corneal and conjunctival damage when ash enters the eyes while wearing SCLs, assuming that SCLs cannot be removed during sudden ashfall. This evaluation was conducted using rabbit eyes.

## 2. Materials and Methods

### 2.1. Animal Preparation and Screening

Eighteen male adult New Zealand White rabbits (2.0–3.5 kg), approximately 1 year old, were used in accordance with the ARVO Statement for the Use of Animals in Ophthalmic and Vision Research. The rabbits were purchased from Japan SLC Inc. (Hamamatsu, Japan). All animals underwent screening eye examinations with a slit lamp and fluorescein and rose bengal staining. Only rabbits with no abnormal findings in their eyes were included in the study.

### 2.2. Volcanic Ash Application and Anesthesia

**Figure 1:**
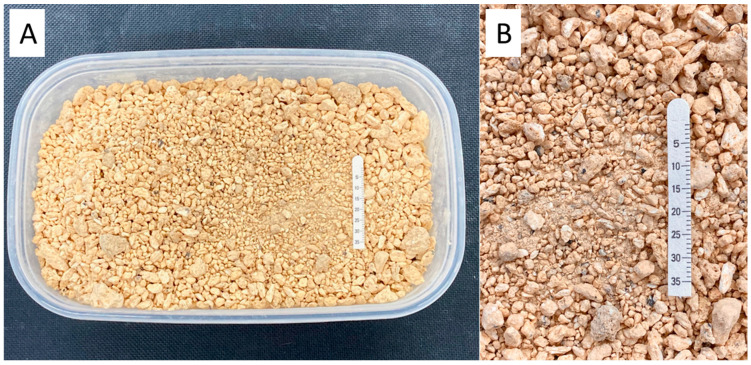
Volcanic ash sourced from Kiso Ontake, Kiso district, Japan. (**A**) displays the image of the volcanic ash in the package upon arrival, while (**B**) is an enlarged photo of the same. The bar represents Schirmer tear test strips, measured in millimeters.

Volcanic ash, sourced as the first pumice from Kiso Ontake Volcano (straddling Kiso district and Gero city, Japan), was obtained from the Quaternary Research Center (Saitama, Japan) (Figure 1). The rabbits were administered general anesthesia as previously described [8], and while under anesthesia, a small spoonful of volcanic ash was sprinkled onto one eye of each rabbit while it was positioned laterally. After the application of volcanic ash, the eye was immediately rinsed with 100 mL of saline solution. The other eye was left untreated and served as a control.

### 2.3. Experimental Animal Groups

The rabbits were randomly divided into three groups, with six rabbits in each group:

(1) Bare eye group: volcanic ash was applied directly to the bare eyes, followed by rinsing with 100 mL of saline solution.
(2) SCL group: Volcanic ash was applied to eyes with SCL worn, followed by rinsing in the same manner as the first group. Air Optix Aqua (Alcon, Fort Worth, TX, USA) was served for this study.
(3) Control group: the rabbits were anesthetized, and only eye rinsing was performed without exposure to volcanic ash.

### 2.4. Evaluation of Ocular Surface Injury

Slit-lamp microscopy with fluorescein and rose bengal staining was performed both before and after treatment with volcanic ash. Fluorescein staining is primarily used to detect corneal epithelial damage, while rose bengal staining is used to identify epithelial damage in the conjunctiva. The ocular surface was evaluated according to a previously reported scoring method [9]. This original van Bijsterveld’s scoring system, conducted with rose bengal staining, rates the nasal conjunctiva, cornea, and temporal conjunctiva on a scale up to 3 points each, totaling a maximum of 9 points. In the present study, we adopted this scoring system for rose bengal staining. For fluorescein staining, we scored only the corneal region, thus the maximum score was 3 points.

### 2.5. Microscopy

After the observations, the animals were euthanized with an overdose of anesthetic, and the cornea and bulbar conjunctiva were excised for observation under light microscopy. The excised tissues were fixed in 4% paraformaldehyde, embedded in paraffin, and then sectioned into thin slices, as previously reported [8]. Subsequently, the corneal tissue was stained with Hematoxylin and Eosin (H and E), while the conjunctival tissue was stained with Periodic Acid-Schiff (PAS). Both were then observed histopathologically using a light microscope.

### 2.6. Statistics

Statistical analysis was conducted with BellCurve for Excel (Social Survey Research Information Co., Ltd., Tokyo, Japan). The statistical significance of the scored values was determined using the Tukey–Kramer method, because it is designed to control the Type I error rate when conducting pairwise comparisons across multiple groups and with a significance threshold set at *p* < 0.05. Values represent the mean ± standard deviation (S.D.). Due to ethical considerations and the constraints of animal experimentation, we were limited in the number of animals we could include in the study.

## 3. Results

### 3.1. Slit-Lamp Microscopy Findings

#### 3.1.1. After Volcanic Ash Application

**Figure 2:**
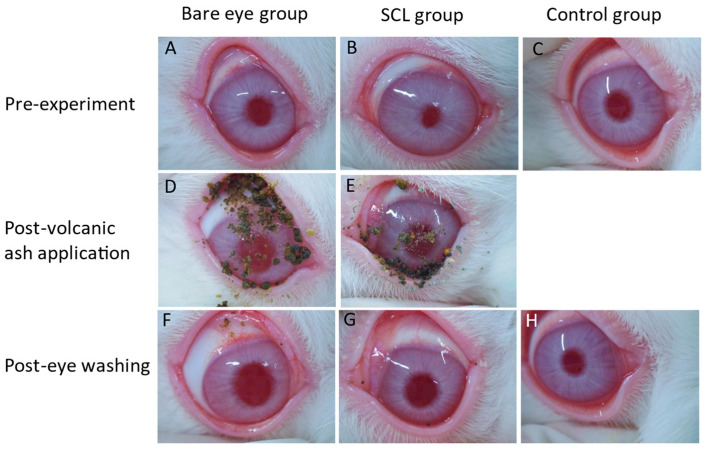
Slit-lamp photographs in bare eye group (**A**,**D**,**F**), SCL group (**B**,**E**,**G**), and control group (**C**,**H**). SCLs were present on eye in (**B**,**E**). Pre-experiment: (**A**–**C**). Post-volcanic ash application: (**D**,**E**). Post-eye washing: (**F**–**H**). In SCL group, SCLs were removed after eye washing (**G**).

In the bare eye group, a large amount of volcanic ash adhered to the cornea and conjunctiva (Figure 2D). In the SCL group, volcanic ash remained on the lens surface and the eye (Figure 2E). The control group remained intact as it was not exposed to volcanic ash.

#### 3.1.2. After Eye Washing

After rinsing with saline solution, in the bare eye group, a small amount of volcanic ash particles remained attached to the cornea and conjunctiva (Figure 2F). In the SCL group, a few volcanic ash particles were shown under the slit lamp (Figure 2G). No changes were observed in the control group (Figure 2H).

#### 3.1.3. Fluorescein Staining

**Figure 3:**
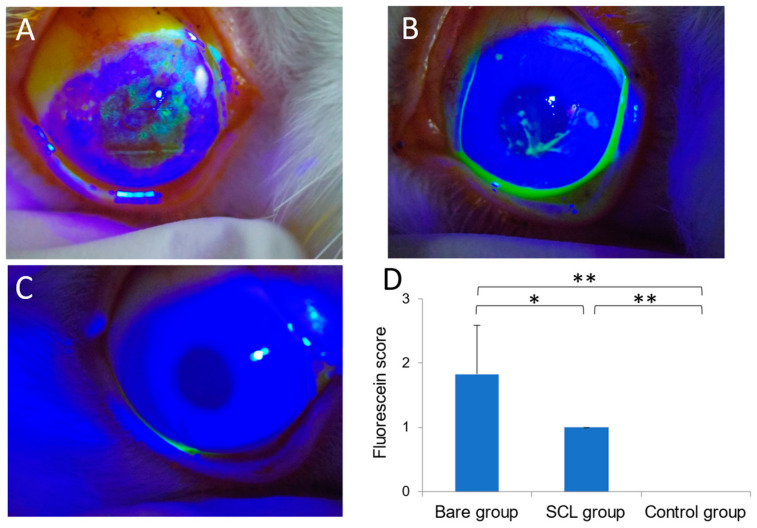
Slit-lamp photographs of fluorescein staining in the bare eye group (**A**), SCL group (**B**), and control group (**C**), along with a histogram of fluorescein scores in Image (**D**). The control group exhibits no staining, resulting in a fluorescein score of zero. Both the bare eye and SCL groups show corneal staining, with the SCL group demonstrating significantly lower staining scores compared to the bare eye group. The mean ± standard deviation of the fluorescein staining scores for each animal group (n = 6) is presented, with significant differences marked by * *p* < 0.05 and ** *p* < 0.01, respectively.

In the bare eye group, more than half of the corneal area showed positive fluorescein staining (Figure 3A). In the SCL group, only a small part of the cornea showed positive fluorescein staining (Figure 3B). No staining was observed in the control group (Figure 3C). While both the bare eye and SCL groups exhibited corneal staining, the staining scores were significantly lower in the SCL group than in the bare eye group (Figure 3D).

#### 3.1.4. Rose Bengal Staining

**Figure 4:**
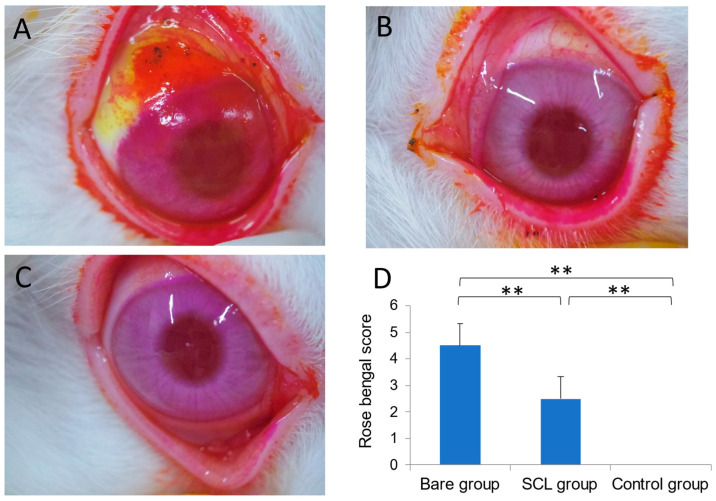
Slit-lamp photographs of rose bengal staining in the bare eye group (**A**), SCL group (**B**), and control group (**C**), along with a histogram of rose bengal scores in Image (**D**). The control group exhibits no staining, resulting in a rose bengal score of zero. Both the bare eye and SCL groups show corneal and conjunctival staining, with the SCL group demonstrating significantly lower staining scores compared to the bare eye group. The mean ± standard deviation of the rose bengal staining scores for each animal group (n = 6) is presented, with significant differences marked by ** *p* < 0.01.

In the bare eye group, more than half of the area of the bulbar conjunctiva and cornea showed positive rose bengal staining (Figure 4A). In the SCL group, only a part of the bulbar conjunctiva and a small area of the cornea showed positive rose bengal staining (Figure 4B). No staining was observed in the control group (Figure 4C). Rose bengal staining was observed in both the bare eye and SCL groups; however, the SCL group shows markedly lower staining scores than the bare eye group (Figure 4D).

### 3.2. Histopathological Findings

#### 3.2.1. Cornea

**Figure 5:**
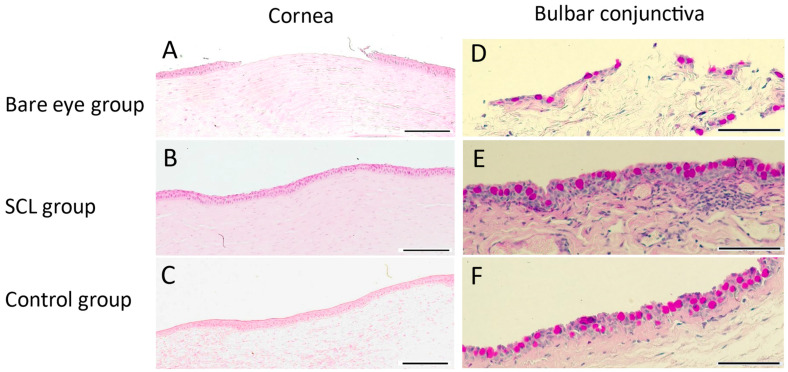
Light microscopic images showing the structure of the cornea in the bare eye group (**A**), SCL group (**B**), and control group (**C**), as well as the bulbar conjunctiva in the bare eye group (**D**), SCL group (**E**), and control group (**F**). The cornea was stained with hematoxylin and eosin, with scale bars at 100 μm; the conjunctiva was stained with PAS, with scale bars at 50 μm. Epithelial damage in both corneal and conjunctival tissues was revealed in the bare eye group.

H and E staining results showed that in the bare eye group, there were defects in the entire corneal epithelium and corneal erosion was observed (Figure 5A). In the SCL group, few defects in the corneal epithelium were observed (Figure 5B). The control group remained intact (Figure 5C).

#### 3.2.2. Bulbar Conjunctiva

Findings from PAS staining indicated that the bare eye group exhibited abnormalities in the conjunctival epithelium and a reduction in PAS-positive cells, which were likely goblet cells of the conjunctiva (Figure 5D). In the SCL group, damage to the conjunctival epithelium was not prominent, and most of PAS-positive cells were preserved (Figure 5E). The control group remained intact (Figure 5F).

## 4. Discussion

Volcanic eruptions occur suddenly, and the effects of volcanic ash on the human body can be both direct and indirect, mediated through environmental impacts. During ashfall, it is recommended that CL wearers remove their lenses to prevent eye damage [2,3]. While this is advisable if there is time before the ash reaches you, in cases where a volcano erupts nearby or a phreatic explosion occurs, people must prioritize their safety and evacuate immediately. In this study, we investigated the effects on SCL wearers compared to those with bare eyes, assuming an emergency situation near a volcanic crater. The results showed, for the first time, that when volcanic ash enters the eyes, those wearing SCLs experienced less corneal and conjunctival damage compared to those with bare eyes (Figure 3, Figure 4 and Figure 5). However, it should be noted that these results are based on animal experiments and may differ from the outcomes in humans.

There are several reports indicating that the closer a location is to a volcano, the greater the impact on the respiratory system and eyes, compared to more distant locations [10,11,12,13,14]. While residential areas are usually located some distance from the crater, leaving little time before the ashfall, there is still generally more time to evacuate than if one were near the crater. Additionally, during activities such as cleaning up volcanic ash after it has fallen, it is essential to protect oneself from ash in a calm and effective manner. In such situations, as previously recommended, CLs should be removed, and masks and goggles should be worn.

The International Volcanic Health Hazard Network (IVHHN) (Durham, England) is responsible for compiling these recommendations. This organization coordinates research and information related to the health impacts of volcanic eruptions [2]. Their work spans a wide range of scientific fields, including volcanology, epidemiology, environmental science, toxicology, public health, and exposure science. The IVHHN’s mission is to identify the health impacts of volcanic emissions and protect exposed communities. To achieve this, the IVHHN collaborates with various organizations, including the International Association of Volcanology and Chemistry of the Earth’s Interior (Canberra, Australia), the New Zealand Geological and Nuclear Sciences Institute (Lower Hutt, New Zealand), and the U.S. Geological Survey (Reston, UA, USA) to produce the “Volcanic Ash Health Effects: A Guide for the Public” [3]. This guide, translated into multiple languages and available for download from the official website, describes the eye symptoms caused by volcanic ash as follows: “Eye irritation is a common health effect as pieces of grit can cause painful scratches in the front of the eye (corneal abrasions) and conjunctivitis. CL wearers need to be especially aware of this problem and leave out their lenses to prevent corneal abrasion from occurring” [3]. This is a crucial message. However, in the event of a sudden volcanic eruption or phreatic explosion, this advice may not apply to CL wearers near the crater. As mentioned above, the immediate priority is to evacuate to safety.

The IVHHN also recommends wearing masks and goggles as a direct protective measure to reduce the impact of volcanic ash on the human body during eruptions. This advice is based on the fact that some people have successfully prevented symptoms by using these protective measures. There are several reports on eye damage caused by volcanic ash. The oldest example is a study conducted in December 1963 that investigated the effects of air pollution caused by the eruption of Irazú Volcano, located about 15 miles east of San José, Costa Rica [10]. In addition to investigating the chemical composition of volcanic emissions, the study examined their effects on the respiratory system and eyes. The volcanic ash reached San José, causing throat and eye irritation among residents. Specific effects on the eyes included eye redness, burning sensations, and eye pain caused by ash particles entering the conjunctival sac. Some residents were able to prevent these symptoms by wearing masks and goggles. This case study from 60 years ago appears to have laid the foundation for the current recommendations on mitigating the health impacts of volcanic ash.

Looking back at the history of CLs, the prototype of the modern hard CL (HCL) first appeared on the market in the 1950s, and the SCL was introduced in the 1970s [15,16]. The first investigation into the effects of volcanic ash on CL wearers was reported by Fraunfelder et al. in 1983 [11]. They studied the impact on the eyes following the eruption of Mount St. Helens in southwestern Washington, USA, in May 1980. The volcanic ash spread across Idaho, Montana, Oregon, and Washington. Among the 1523 patients surveyed by 322 ophthalmologists, there were 1230 cases (80.7%) of foreign body sensation in the eyes, 753 cases (49.4%) of conjunctivitis, 126 cases (8.3%) of foreign bodies in the conjunctiva, and 123 cases (8.1%) of foreign bodies in the cornea, with nearly 20% requiring foreign body removal. The study indicated that HCL wearers experienced more problems than SCL wearers. Specifically, the ash particles scratched the lenses, entered beneath them, and caused corneal abrasions, leading to a significant number of complaints about foreign body sensation. In contrast, SCL wearers reported fewer symptoms of eye irritation. This study highlights that the type of CLs worn can significantly influence the effects of volcanic ash on the eyes.

As of the 2020s, 94.5% of the world’s CL wearers use SCLs [4]. Therefore, in the event of a sudden eruption occurring close to the crater, based on our findings that SCL wearers experience less corneal and conjunctival damage than those with bare eyes, we recommend that SCL wearers continue wearing their lenses and prioritize evacuation from the dangerous area. If the lenses are removed, it could become even more difficult to evacuate safely due to impaired vision, and their hands might also be contaminated with volcanic ash. In this study, corneal epithelial defects were observed in bare eyes, which were demonstrated in vivo through fluorescein staining and in vitro through H&E staining under a light microscope. Similarly, conjunctival epithelial damage was confirmed in vivo with rose bengal staining and in vitro with PAS staining. Generally, when a foreign object enters the eye, the pain can be considerable, and if corneal or conjunctival epithelial defects occur, it would be difficult not only to evacuate to a safe place but also to walk, as keeping the eyes open would be nearly impossible.

In some regions, such as Japan’s Sakurajima, the average number of volcanic eruptions per year is about 200, with some years experiencing as many as 996 eruptions [12]. A study investigating eye damage among elementary and junior high school students living in this region found that those living closer to the volcano, within 2.5 miles, experienced more eye injuries and irritation. These issues were attributed to the mechanical irritation caused by volcanic ash. Additionally, there have been reports of health effects caused by volcanic fog (vog) resulting from volcanic eruptions. For example, a study in Hawaii involving 30 patients with conjunctivitis due to vog found that the main causes were irritation and allergic reactions triggered by chemicals in the vog, such as sulfur dioxide and sulfuric acid aerosols [13]. Other studies have also noted that those involved in cleaning up volcanic ash often experience eye and respiratory symptoms. This reinforces the IVHHN’s recommendation to wear goggles or eyeglasses to prevent eye injuries. However, unfortunately, many existing reports did not include CL wearers in their investigations.

Aside from volcanic ash, there are reports highlighting the benefits of wearing SCLs when exposed to harmful substances such as air pollution, organic solvents, acids, and alkalis, particularly in industrial and chemical workplaces [6,7,17]. Summarizing these findings, SCLs provide protection to the ocular surface in the early stages of exposure, but prolonged wear may lead to adverse effects. Therefore, if these harmful substances enter the eyes, it is recommended to remove the lenses as soon as possible and immediately rinse the eyes. Conversely, in military environments where sandstorms may occur, there have been reports of eye injuries among CL wearers in situations where the lenses cannot be removed immediately [18,19]. In such cases, sand particles can become trapped under the lenses, leading to corneal and conjunctival damage. As noted in these papers, prolonged corneal epithelial damage due to these reasons can potentially lead to the development of corneal infections. A previous report has also emphasized the importance of wearing goggles, particularly in desert, temperate, and underwater environments, depending on the conditions in which personnel are deployed [19].

Another important caution emerged from our experiment. We simulated the scenario where volcanic ash fell on rabbit eyes while wearing SCLs. Even after removing the SCLs and rinsing with saline solution, volcanic ash particles remained on the cornea and conjunctiva, and rinsing alone was not sufficient to remove all of them. Therefore, even in the case of human eyes, if volcanic ash enters the eyes and eye pain or a foreign body sensation persists after removing the SCLs, it is strongly recommended to seek immediate ophthalmic care to have any remaining particles removed. This is because corneal abrasions caused by volcanic ash particles under the SCL, as well as residual foreign bodies in the conjunctiva, are suspected.

Our study focused on the eye damage that could occur during sudden volcanic ashfall at close range, necessitating urgent evacuation. However, there are also several reports that have examined the chronic effects of volcanic ash on the eyes [12,20]. For instance, Sakurajima in Japan is an active volcano with frequent eruptions, and due to its proximity to a large city, many residents are chronically exposed to ashfall. In studies conducted near this volcano, it was found that the concentration of suspended particulate matter in nearby cities often exceeded national environmental quality standards. Despite prolonged exposure to volcanic ash, there was no significant difference in the prevalence of non-specific respiratory diseases, and respiratory symptoms were not substantially higher compared to other regions. On the other hand, ocular symptoms such as itching, conjunctival injection, and tearing were reported. However, a study that observed the impact of volcanic eruptions on the eyes of schoolchildren over ten years did not directly compare the prevalence of eye symptoms to other regions [12]. It is important to mention that these symptoms were observed even in asymptomatic children, indicating that volcanic ash exposure can cause subclinical or mild ocular irritations that might not be immediately noticeable to those affected. The study emphasizes that these findings were more prevalent in areas closer to the volcano, where exposure to volcanic ash was higher. These ocular symptoms were primarily related to the mechanical irritation caused by the ash particles. Furthermore, the potential for chronic inflammation of the conjunctiva was also discussed. The paper also noted that no vision-threatening complications were found, and symptoms were effectively managed with treatments such as eye drops. Furthermore, these studies did not compare CL wearers with those wearing eyeglasses or those with no corrective eyewear. To the best of our knowledge, no such attempts have been made in the existing literature. This could be a potential area for future research.

Based on the findings of this study and previous reports, it is considered important that SCL wearers, if they feel any abnormalities in their eyes during volcanic ashfall, move to a safe location, remove their SCLs, and promptly seek ophthalmic care.

## 5. Conclusions

In the event of a sudden volcanic eruption, SCL wearers should continue wearing their lenses while prioritizing evacuation from the dangerous situation. After reaching a safe location, it is advisable to follow the IVHHN’s recommendations and wear goggles or eyeglasses. Furthermore, if any eye discomfort or abnormalities are experienced after removing the SCLs, seeking immediate ophthalmic care is recommended.

## Data Availability

All data are provided in the main text.
